# Correlation between Ca Release and Osteoconduction by 3D-Printed Hydroxyapatite-Based Templates

**DOI:** 10.1021/acsami.4c01472

**Published:** 2024-05-25

**Authors:** Mohamad N. Hassan, Ahmed M. Eltawila, Samih Mohamed-Ahmed, Wessam M. Amin, Salwa Suliman, Sherif Kandil, Mohammed A. Yassin, Kamal Mustafa

**Affiliations:** † Centre for Translational Oral Research (TOR), Department of Clinical Dentistry, Faculty of Medicine, University of Bergen, Årstadveien 19, Bergen 5009, Norway; ‡ Orthopedic Clinic, Haukeland University Hospital, Helse Bergen, Haukelandsveien 28, Bergen 5021, Norway; § Department of Materials Science, Institute of Graduate Studies and Research (IGSR), Alexandria University, El-Shatby, Alexandria 21526, Egypt; ∥ Department of Dental Biomaterials, Faculty of Oral and Dental Medicine, Delta University for Science and Technology, Coastal International Road, Gamasa 11152, Egypt; ⊥ Biomaterials Section, Department of Clinical Dentistry, Faculty of Medicine, University of Bergen, Årstadveien 19, Bergen 5009, Norway

**Keywords:** PLATMC, calcium phosphates, blending, printability, rabbit, calvarial bone defect, bone regeneration

## Abstract

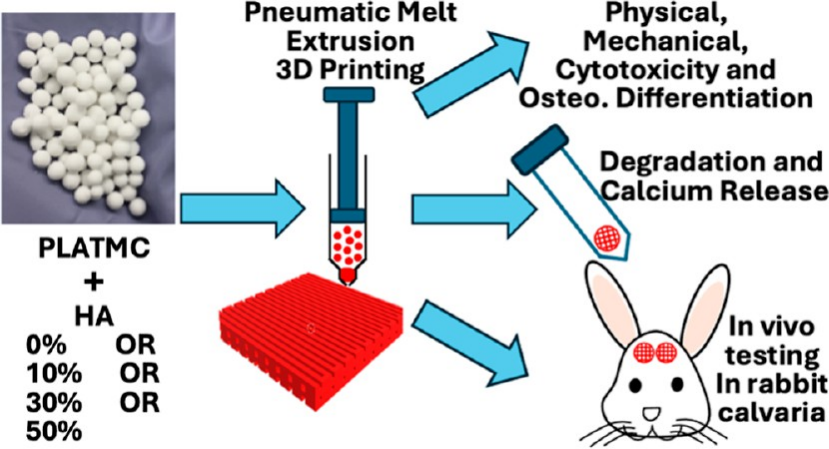

The application of hydroxyapatite (HA)-based templates is quite often seen in bone tissue engineering since that HA is an osteoconductive bioceramic material, which mimics the inorganic component of mineralized tissues. However, the reported osteoconductivity varies in vitro and in vivo, and the levels of calcium (Ca) release most favorable to osteoconduction have yet to be determined. In this study, HA-based templates were fabricated by melt-extrusion 3D-printing and characterized in order to determine a possible correlation between Ca release and osteoconduction. The HA-based templates were blended with poly(lactide-*co*-trimethylene carbonate) (PLATMC) at three different HA ratios: 10, 30, and 50%. The printability and physical properties of the HA templates were compared with those of pristine PLATMC. In vitro, osteoconductivity was assessed using seeded human bone marrow-derived mesenchymal stem cells. A mild rate of Ca release was observed for HA10 templates, which exhibited higher mineralized extracellular matrix (ECM) secretion than PLATMC at 14 and 21 days. In contrast, the high rate of Ca release exhibited by HA30 and HA50 templates was associated with reduced osteoconduction and impeded mineralized ECM secretion in vitro. Similar results were observed in vivo. In the calvarial defect model in rabbit, PLATMC and HA10 templates exhibited the highest amount of new bone formation, with obvious contact osteogenesis on their surfaces. In contrast, HA30 and HA50 exhibited distant osteogenesis and reduced amounts of new bone ingrowth. It is concluded that HA-based templates are osteoconductive only at low rates of Ca release.

## Introduction

Bone is a mineralized tissue, with cellular components of around 10%. Secreted extracellular matrix (ECM) accounts for most of the bone tissue volume. The major component of ECM is the mineralized inorganic matrix, formed by the precipitation of hydroxyapatite (HA) crystals.^1^ To generate bone on implanted templates, osteoconduction is essential to support the recruitment and migration of differentiating osteogenic cells to the implant surface.^2^ This facilitates contact osteogenesis, i.e., the secretion of mineralized ECM, with new bone formation directly on the implant surface.^3^ The desired osteoconduction is achieved either by inherent or engineered physicochemical characteristics at the material/tissue interface or by the presence of bioactive motifs or molecules intended to be released into the local tissues of the host.^4^

Calcium phosphates (CaP), including HA and β-tricalcium phosphate (β-TCP), are abundant in native bone and have been used extensively as implantable osteoconductive biomaterials.^5^ This osteoconductive potential of CaP is related to their degradation and ion release, which can be regulated by customizing the chemical composition, surface topography, and pore geometry of the implanted templates.^6^ HA consists of calcium (Ca) and phosphate (P) ions in a crystalline form and possesses high osteoconductive and potential osteoinductive properties.^7^ It is regularly used in bone grafts and templates, alone or blended with different polymers. Porous HA implants, powders, and coatings on metallic prostheses have been routinely used to provide osteoconductive fixation with bone.^8,9^

It was hypothesized that when implanted in vivo, the concave micropore geometry of conventional HA templates would concentrate bone-forming molecules, such as bone morphogenetic proteins (BMPs), and stimulate angiogenesis, which induces bone formation.^10^ Moreover, inconsistent bone regeneration outcomes have been reported for different forms of HA templates, such as foamed microporous and 3D-printed orthogonal-patterned templates. The concave micropore geometry and specific surface area were considered to be the key factors underlying greater amounts of bone formation by foamed HA templates than 3D-printed HA templates.^11,12^

On the contrary, this concave micropore geometry exhibited no advantage over the orthogonal-patterned pores when tested on titanium (Ti) cylinders in vivo.^13^ Other reports have disclosed no osteoconductive advantages of HA-based and β-TCP templates over plain polymers in vitro, or when implanted in calvarial defects in the rabbit, except when functionalized with BMPs.^14^ Nevertheless, none of the aforementioned studies investigated whether the discrepancies in the amount of new bone regeneration might be attributable to variations in Ca release from the HA templates.^11,12,14^ In other studies, Ca release rates were measured after only a few hours.^6^

Indeed, the optimal Ca concentration may vary according to cell type and cellular stage, in order to achieve the desired in vitro osteoconductivity,.^15,16^ Reduced osteoblast cell proliferation, lower osteoblastic gene expression, and impeded ECM secretion were observed in vitro at nano HA particle concentrations higher than 25 μg/mL,^17^ and inhibited cell differentiation was associated with the presence of high concentrations of exogenous Ca in vitro.^18^ Thus, to date, this question has not been extensively investigated and the exact mechanism of HA osteoconductivity and the role of Ca concentration have yet to be clarified.

The main aim of this study was to explore a possible correlation between Ca release over time by 3D-printed HA-based templates and the corresponding degree of osteoconduction, initially in vitro and then in vivo, in calvarial bone defects in the rabbit. Submicron-sized HA was used due to its expected faster degradation and closer biophysical characteristics to natural bone apatite.^19^ This was blended with poly(lactide-*co*-trimethylene carbonate) (PLATMC), recently reported to have good mechanical, degradation, and osteoconductive properties.^20^ The templates were 3D-printed using the melt-extrusion method and Ca release was monitored in vitro for up to 100 days.

## Experimental Section

### Preparation of HA Blends

**Figure 1:**
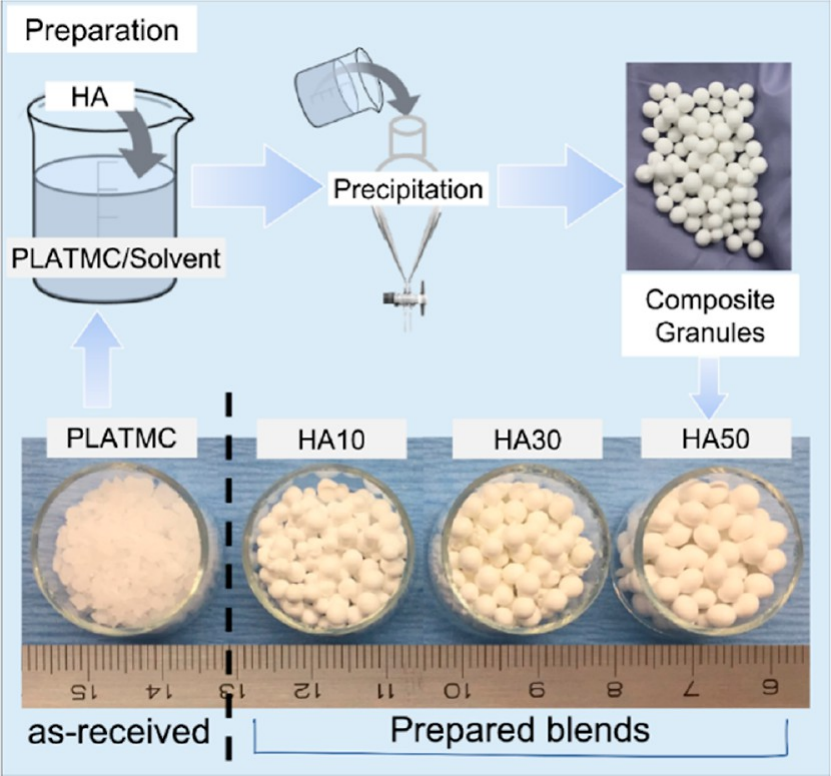
Sketch of the blending process of PLATMC (as received) with HA and its precipitation in granules. The resulting HA blends are pictured, in the form of granules ready for further processing (3D-printing).

HA (<200 nm particle size, Sigma, USA) was selected because it is easily blended and printed with PLATMC. Using a recently developed physical suspension method, PLATMC was blended with HA at 10, 30, or 50 (w/w)%.^21^ Dimethyl sulfoxide (DMSO) was used as a solvent. PLATMC was dissolved in DMSO at 80 °C and stirred for 2 h. HA was dispersed in DMSO and sonicated for 30 min (three times). The dispersed HA was added to the PLATMC solution and stirred for 1 h. The PLATMC/HA solution was then precipitated (dropwise) into distilled water (dH_2_O). The precipitated beads were washed in distilled water (1 h), filtered, and dried (1 h). Finally, the beads were frozen overnight and freeze-dried for 24 h before being printed (Figure 1).

### Printing of HA Blends

PLATMC and HA blends were printed using a pneumatic melting-extrusion printer (3D-Bioplotter, Manufacturer Series, EnvisionTEC, Germany) with an 0.4 mm diameter nozzle, at adjustable printing parameters, as shown in Table 1. Each layer was printed with a fixed interstrand distance (0.3 mm) and orientation between layers at 0/90°. The printed sheets (four layers, 30 mm × 30 mm) were then punched out to the specific diameter for each test.

**Table 1:** Printing Parameters for PLATMC and HA Blends

	(bar)	temperature^a^ (°C)	printing speed (mm/s)	feed/syringe (g)	printing time (total feed) (min)
PLATMC	8.0	195	2.0–5.0	3.0	85
HA10	8.0	200	4.5	2.7	85
HA30	8.0	205	2.5	3.1	105
HA50	8.0	210	2.5	3.1	70

a Polymers were preheated for 15 min, at 15–25 °C above the actual recorded temperature.

### Sterilization for Biological Assessment

All printed templates used for biological characterization (in vitro and in vivo) were sterilized in ethanol (70%, 10 min, twice) under sonication. The ethanol was then aspirated in a biosafety cabinet. The samples were washed twice in sterile PBS, dried, and then exposed to UV light for 1 h,^22^ before being packed in sterile bags and stored at 4 °C until use.

### Physical Characterization

#### Printability and Yield Calculations

The printing-yield and density of the printed sheets per each group were calculated to compare the processing parameters. The printing-yield was calculated according to the following equation
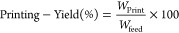
where *W*_print_ is the total weight of printed sheets per each printing-run and *W*_feed_ is the gross weight of the feed materials added to the printing cartridge for each specific printing-run. The weight of the printed groups was recorded to calculate their density (g/cm^3^) as follows: density = *W*_print_/*V*_print_, where W_print_ is the weight of printed templates in grams, while *V*_print_ is their calculated geometric volume.

#### Thermal Analysis

Thermal analysis of 3D-printed samples was carried out by a thermogravimetric analyzer (TGA-50/50H, Shimadzu, Japan) from room temperature (RT) to 450 °C, at a heating rate of 10 °C/min.

#### Surface Characterization and Wettability

The surface roughness of the printed templates was investigated using a roughness measurement tester (Perthometer M2, Mahr GmbH, Germany). The surface morphology of the printed templates was viewed in a scanning electron microscope (Phenom XL Desktop, Thermo Fisher) at 10 kV by the backscatter detector. The templates were dried and then sputter coated with gold–platinum (around 50 Ångstrom thickness). The surface atomic contents were identified by energy-dispersive X-ray (EDX) analysis, at a working distance of 5.5 mm, for the presence of Ca and P ions.

A water contact angle assessment was applied (at RT) to the prepared 3D-printed HA blends (n = 5) to determine their wettability (Contact Angle Goniometer model 90, CA Edition, ramé-hart, USA). Water (3 μL) was dropped onto the surface of each sample, and the average contact angle was recorded (for triple measurements) at various positions on the surface.

#### Mechanical Characterization

Dumbbell-shaped samples (shaft dimensions = 17.5 mm × 4.5 mm × 1.5 mm) were printed according to ASTM-D638, to test the tensile properties of each group. The tensile stress and Young’s modulus (*n* = 5) were tested using a universal tensile testing machine (MTS, 858 mini Bionix II instrument, Eden Prairie, MN, USA), at RT, at a tensile displacement rate of 3 mm/s.

#### In Vitro Mass Loss (Degradation)

In vitro degradation was assessed by recording the mass loss of dried samples and monitoring the changes in morphology over time.^23^ 3D-printed samples (Ø = 8 mm, *n* = 4/time point/group) were weighed precisely (*W*_o_) and then added in PBS (900 μL/sample) to 48-well plates. The samples were marked to be matched with their individual mass change (specific for each sample), and the plate was sealed (parafilm) and incubated under shaking (37 °C, 100 rpm). The PBS was replaced every 5 days, up to 100 days. The mass change was recorded at 15, 30, 60, and 100 days, where the samples were washed (dH_2_O, three times) dried at (37 °C, 4 h) and then freeze-dried (48 h). The mass loss (%) was calculated according to the following equation
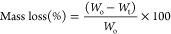
where *W*_o_ is the original weight of each template before immersion in PBS, and *W*_t_ is the dry weight recorded at each time point. Representative tested samples after 60 and 100 days were then sputter-coated (with gold–platinum) and examined by scanning electron microscopy (SEM) at 10 kV by a secondary electron detector to detect signs of surface degradation.

#### In Vitro Ca Release

The Ca released by 3D-printed HA blends (*n* = 4) was assessed after immersion in PBS (1 mL/sample, 37 °C) under shaking (100 rpm).^24^ The PBS was aspirated at 1 h, then at 1, 2, 3, 4, 5, 7, 9, 15, 30, 50, 80, and 100 days, and replaced by freshly prepared PBS. Printed PLATMC samples were recorded as the baseline. The Ca concentration in aspirated PBS was quantified by Calcium Assay Kit (Colorimetric) (ab102505, Abcam, UK) compared to a standard Ca concentration, according to the manufacturer’s recommendations, at absorbance = 575 nm. To calculate the amount of Ca released per unit mass of template (μg/g), the Ca released (quantified values from the standard curve in μg) was then multiplied by the dilution factor and divided by the average weight of the samples. The data were presented as Ca concentration, released at each time point and as the cumulative total Ca released up to 100 days.

### In Vitro Osteoconduction by 3D-Printed HA Blends

#### Cell Seeding

Human bone marrow-derived mesenchymal stem cells (hBMSCs) were isolated from bone marrow aspirates from the anterior iliac crest of 8–14 year old patients, undergoing iliac crest surgery for cleft lip and palate repair at the Department of Plastic, Hand and Reconstructive Surgery, National Fire Damage Center, Bergen, Norway. Informed parental consent was obtained. Ethical approval for this study was granted by the Regional Committee for Medical and Health Research Ethics (REK) in Norway (ref. no. 2013/1248/REK sør-øst C). The isolated hBMSCs were characterized and kept frozen in liquid nitrogen (passage 2) as previously documented.^25^ The cells were thawed in α-MEM, expanded, and seeded onto the 3D-printed templates at passage 4. The in vitro osteoconduction assessment was repeated twice, using two different donor cells. The seeding efficiency of hBMSCs on PLATMC and HA blends was calculated after seeding, 8–12 h after incubation at 37 °C in 5% CO_2_. The seeded templates were transferred to another plate, and the remaining cells, attached and suspended cells per each well, were collected in 1.5 mL tubes (Eppendorf safe-lock), centrifuged, and resuspended in 100 μL α-MEM, stained (trypan blue 4%) and counted. The seeding efficiency was calculated using the following equation
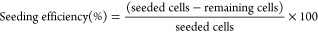


#### Live/Dead Staining Assay

Using a LIVE/DEAD Viability/Cytotoxicity kit for mammalian cells (Invitrogen), a stock solution of PBS containing ethidium homodimer-1 (red, 2 μL/mL) and calcein AM (green, 1 μL/ml) was prepared and vortexed. Seeded templates (7 and 14 days) were washed twice with PBS to remove remnant medium and serum. The prepared working solution was then added directly to cover the cells, incubated under shaking (30 min, RT). The cells were then observed directly in a fluorescence microscope (Nikon Eclipse Ti, Tokyo, Japan) at an excitation/emission equal to 494/517 nm (calcein AM) and 528/617 nm (ethidium homodimer-1). At least 10 captured images were stacked at a 10 μm z-distance.

#### AlamarBlue Assay

Cell viability and mitochondrial activity were quantified using the reducing power of living cells to alamarBlue reagent (alamarBlue HS, Invitrogen, Thermo Fisher Scientific, USA). The reagent was added directly to cells in culture medium (1:10 ratio) and incubated with protection from direct light (4 h, 37 °C), and the fluorescence was read immediately (in duplicate, excitation/emission at 560/590 nm). The results were calculated by subtracting the background fluorescence.

#### Proliferation Assay (DNA Quantification)

The DNA of cells attached to 3D-printed templates was quantified using a Quanti-iT PicoGreen dsDNA assay kit (Invitrogen, Thermo Fisher Scientific, USA), in cell lysis solution (300 μL, 0.1% Triton X-100). The samples (*n* = 5) were frozen (−80 °C), thawed (twice), cut into pieces, added to 1.5 mL tubes with the lysate solution, sonicated (10 min on ice), vortexed (1200 rpm, 10 s), and centrifuged (2 min, 10,000 rpm). The supernatant (50 μL) was aspirated (in duplicate) and added to diluted PicoGreen dye, where the fluorescence intensity was measured (excitation/emission = 485/520 nm) and compared to a standard curve obtained by serial dilution of a known concentration of DNA (μg/mL).

#### Alkaline Phosphatase Activity

P-nitrophenyl phosphate (pNPP, Sigma) was added (1:1) to lysate solution to measure the secreted ALP activity, as an indicator of osteogenic ECM secretion by the seeded cells (*n* = 5). Absorbance was measured at 405 nm at different time points (5, 10, and 15 min), and the results were normalized to the quantified attached cell number determined by the proliferation assay.

#### Osteogenic Gene Expression

To analyze the osteogenic gene expression of seeded cells on 3D-printed templates, RNA was extracted (7 and 21 day samples, *n* = 5) using a Maxwell 16 LEV simplyRNA kit (Promega, Madison, WI, USA) and measured by a Nanodrop ND-1000 Spectrophotometer (Nanodrop Technologies, Wilmington, DE, USA). cDNA was synthesized using a High-Capacity cDNA Reverse Transcription Kit (Applied Biosystems, Foster City, CA, USA), and SimpliAmp Thermal Cycler (Applied Biosystems). To perform a real-time quantitative polymerase chain reaction, TaqMan Fast Universal PCR Master Mix (Applied Biosystems) was added to synthesized cDNA and put into a StepOne RT-PCR System (Applied Biosystems) to detect the gene expression of the osteogenesis-related human genes. Samples were assessed in duplicate, and the amplification efficiency of different genes was determined relative to an endogenous control: glyceraldehyde-3-phosphate dehydrogenase (GAPDH) gene. Data were analyzed by the 2^–ΔΔCT^ method, where ρρCt = ρCt gene - ρCt control, and ρCt represents the difference in threshold cycle value (Ct) between the Ct gene and the Ct housekeeping gene (GAPDH). The relative transcript levels were presented as fold change (in Log scale) relative to the control group (PLATMC).

#### ECM Characterization by SEM

To observe secreted ECM and deposited calcification on 3D-printed HA blends at 14 days, seeded samples were fixed in glutaraldehyde solution (2.5%, pH 7.2) for 30 min and then dehydrated through a graded series of ethanol solutions (70, 80, 95, and 100%) for 10 min each. Dried samples were mounted on aluminum sample holders, sputter-coated with gold–platinum, and examined by SEM (at 10 kV, secondary electron detector). The ECM contents were examined for the presence of Ca and P ions, identified by EDX, at a working distance of 5.5 mm.

#### Alizarin Red Staining

The mineral deposits resulting from osteogenic differentiation of the seeded cells were assessed at 21 and 28 days by Alizarin red staining (2% solution, pH = 4.2, RT, 10 min). The samples were then washed repeatedly, dried overnight in 70% ethanol, and examined in a stereomicroscope (LEICA M205 C, Germany). Representative images were captured by the mounted microscope camera. To quantify the stained mineralization, the dye was extracted using cetylpyridinium chloride: 300 μL (100 mmol) was added to each sample (RT, 4 h) and then aspirated and quantified in duplicate by a microplate reader at OD = 544 nm.

### Osteoconduction Assessment In Vivo

#### Implantation of 3D-Printed HA Blends in Calvarial Bone Defects

The animal experiment protocol was reviewed and accepted by the Institutional Animal Care and Use Committee, Alexandria University, approval no. AU14-191013-2-5. The 3D-printed PLATMC and HA templates were implanted in induced calvarial bone defects in New Zealand white (NZW) rabbits. Bilateral bone defects (Ø = 9 mm) were created by a trephine bur, and the prepared templates of PLATMC and HA blends were implanted (Figure S2). Empty defects served as negative controls. In total, 20 skeletally adult male NZW rabbits were used, divided into two groups at each time point (4 and 8 weeks, *n* = 4/group/time point). For the surgical implantation, the rabbits were anesthetized (xylazine IM, 10 mg/kg, and ketamine IM, 25 mg/kg), the surgical site was shaved and wiped with Povidone-iodine, an incision line (3–4 cm long) was made on the crest of the sagittal suture, and the skin and the periosteum were elevated. After the bone defects were trephined, the 3D-printed PLATMC and HA templates were randomly assigned to the induced defects and the surgical wound was sutured in layers. Topical antibiotic (Gentamicin) was applied to cover the surgical wound to prevent contamination, and a painkiller (diclofenac sodium, IM, 5 mg/kg) was administrated for the first 3 days postsurgery. As indications for humane euthanasia of an animal before the experimental objective had been achieved, the following criteria were applied: significant signs of weight loss, impaired mobility, or extended surgical site inflammation. The rabbits were sacrificed at the due time points, and the collected bone samples were fixed in 4% paraformaldehyde for further analysis.

#### Microcomputed Tomography

After fixation, the collected samples were assessed by microcomputed tomography (μCT) to determine the amount of bone formation within the implanted templates. Samples were scanned using the SkyScan 1172 μCT imaging system (SkyScanVR v.1.5.23, Kontich, Belgium) at 40 kV voltage and 250 mA current at 10 μm resolution. The raw images of the samples were reconstructed using a cone beam reconstruction algorithm. The samples were then prepared for histological examination and histomorphometric (quantitative) analysis.

#### Undecalcified Histological Processing and Histomorphometric Analysis

Calvarial samples with implanted 3D-printed PLATMC and HA blends were gradually dehydrated (ethanol 70% up to 100%), cleared (by Xylene) and processed undecalcified for plastic embedding (PMMA, Technovit 9100, Kulzer, Germany), and trimmed and sectioned by a high-precision cutoff machine (Accutom-100, Struers – Denmark). To obtain five serial sections (around 560 μm apart, including the thickness of the diamond disc) for histomorphometric analysis, the first cut (0.100 mm/s) was sited at the coronal middle third of the bone defect, parallel to the grid structure of the implanted templates. Plexi-glass slides were glued onto the cut surface using transparent, low-viscosity, instant adhesive (Loctite 424, Henkel Adhesive Technologies, Bromma, Sweden), avoiding the creation of air bubbles. Sections were ground (up to 40 μm thickness) and polished (on a polishing cloth, MD Mol, Struers). The polished samples were stained by Toluidine blue (1% solution +0.5% Borax, pH = 10, for 10 min, then washed with dH_2_O) and acid Fuchsin (2% solution, pH = 6, for 20 min), washed (70% ethanol), and left to dry. The sections were then scanned with a light microscope to capture images of the area of interest, followed by histomorphometric analysis using software (NIS-Elements, Nikon, Japan). The defect area (region of interest: ROI) was marked, from both edges of the template/defect and the available defect area was calculated as follows: Available defect area (AA) = total ROI – template area. The sum of new bone ingrowth area (BA) within the defect was calculated, and the data were represented as BA/AA (%). The mean of at least three sections in each sample was calculated, and the mean of each group (*n* = 4) was plotted in bar charts.

#### Decalcified Histological Processing and Bone Contact Analysis

Representative samples were then depolymerized (xylene/chloroform solution, 1:1, for 3–5 h) under shaking, rehydrated, and embedded in xylene (twice) and then in an ethanol series (from 100% up to 70%). The bone samples were then decalcified in EDTA solution (10%, refreshed twice/week, for 4 weeks), then dehydrated, and embedded in paraffin. The samples were then sectioned (5 μm sections), stained with Masson’s Trichrome staining, and observed under bright-field microscopy.

### Data Presentation and Statistical Analysis

Prism software (GraphPad software, San Diego, CA, USA) was used for statistical analysis. The results were expressed as group average ± standard deviations. One-way analysis of variance (ANOVA) was used to detect significant differences in comparisons involving only one time point. For multiple group comparison at different time points, two-way ANOVA was applied. The null hypothesis was rejected at *p*-value <0.05, and Tukey’s post hoc adjustment was used in all data comparisons. All physical and in vitro tests were repeated twice, if not stated otherwise, and the involved sample size was indicated in the methods section of each assay.

## Results

### HA Blends: Successful Preparation and Printability

**Figure 2:**
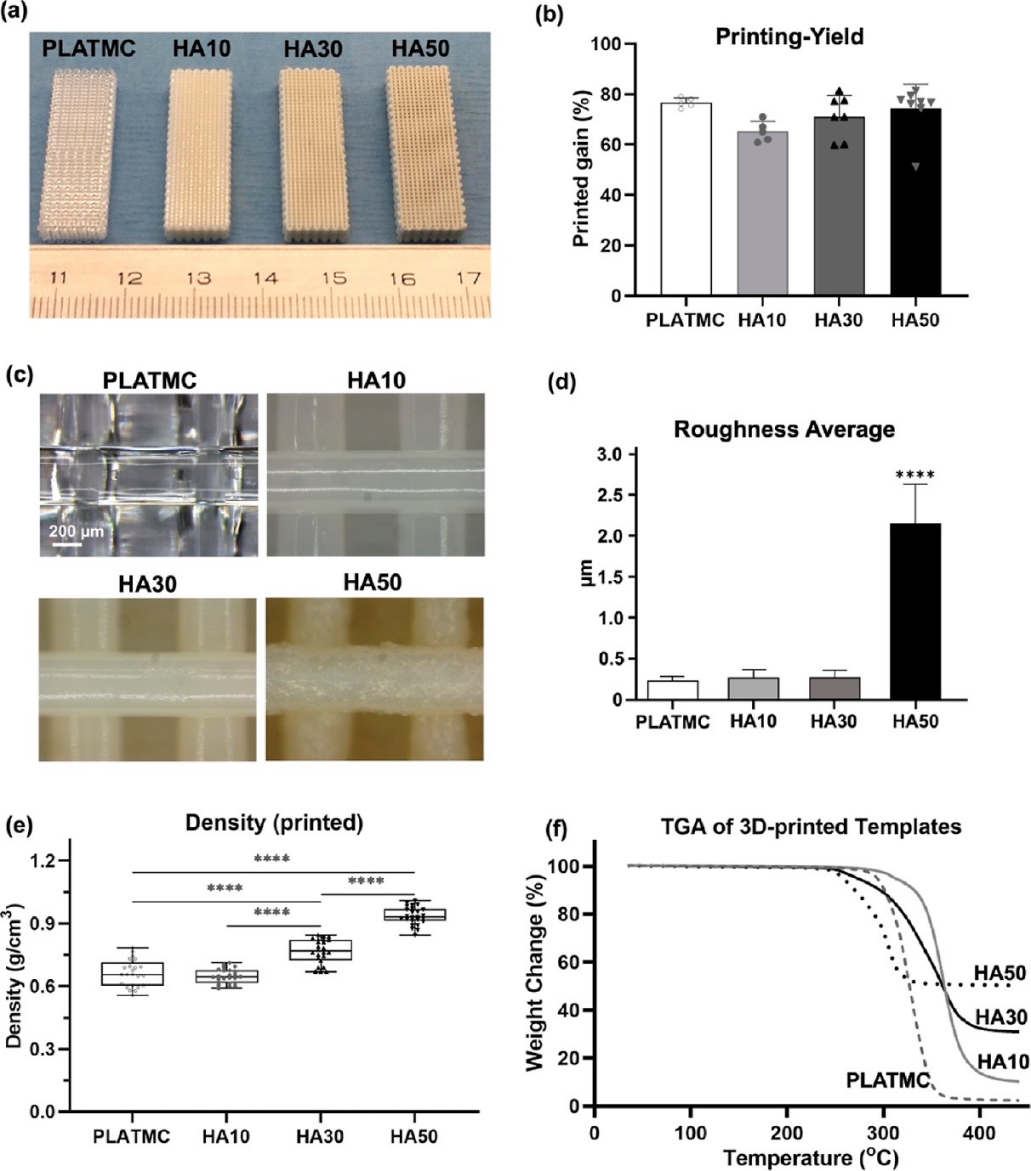
Printability of 3D-printed HA blends and their physical characterization. (a) Macroscopic photographs of the printed templates; (b) column chart of the printability yield; (c) photomicrographs of the printed templates, comparing their strand structure; (d) column chart of the average roughness measurements; (e) density of the printed templates, and (f) TGA characterization to confirm the actual HA content of the templates after the decomposition of polymer contents (up to 400 °C). The statistical significance between the groups is marked with asterisks (*) at *p* < 0.05; ****p < 0.0001.

The preparation of HA blends at the required ratios, 10, 30, and 50% (w/w % HA) with PLATMC, and their printability through direct melt-extrusion revealed homogeneous and well-printed structures macroscopically (Figure 2a). In addition, no intergroup differences in printing-yield were observed among the four groups (Figure 2b). Microscopically, the printed microstructures were homogeneous with the same strand width (Figure 2c), with no variation in the surface roughness among the groups, except for HA50 group templates that presented obvious average surface roughness (2.15 μm), which was eight times higher than the values at PLATMC, HA10, and HA30 printed groups (Figure 2d). The density of the 3D-printed structures increased significantly with increasing HA ratio (Figure 2e) due to the included HA, while thermogravimetric analysis (TGA) results revealed that the percentages of HA within the 3D-printed structures were as accurate as during the HA blending (Figure 2f).

### Printed HA Blends Exhibit Ca-Enriched Surfaces but Decreased Tensile Properties

**Figure 3:**
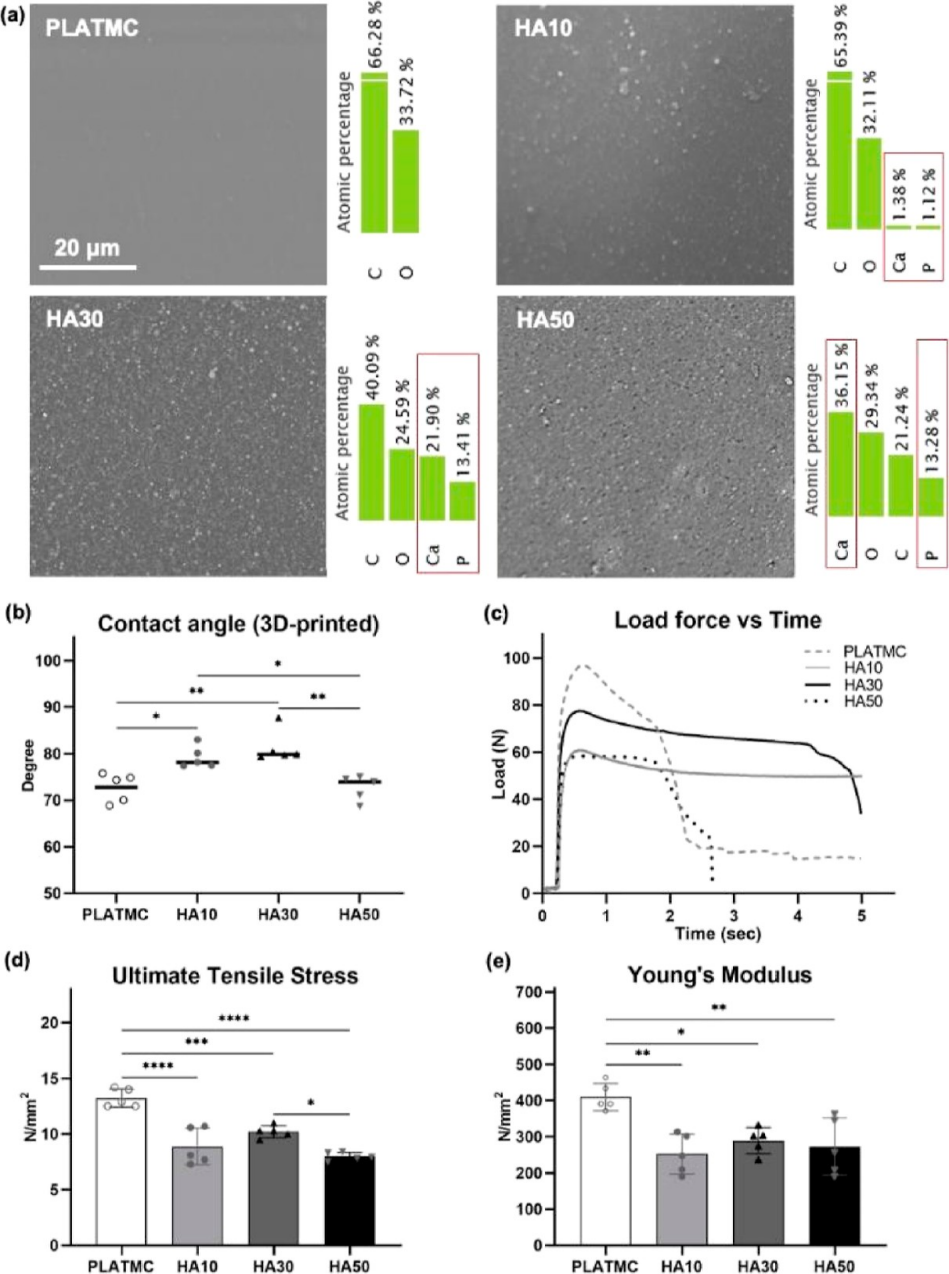
Surface and mechanical characterization of 3D-printed HA blends including: (a) SEM micrographs at backscatter mode, with EDX showing the atomic percentage of Ca and P presented on the surface; (b) mean contact angle measurements showing the wettability of the 3D-printed templates; and (c–e) tensile mechanical properties of templates: (c) load force vs time curves, and (d,e) column charts to the calculated ultimate tensile stress, and Young’s Modulus, respectively. Significance between groups is marked with asterisks (*) at *p* < 0.05.

SEM of the surfaces of the 3D-printed HA blends showed minor submicron roughness on HA10 compared to that on PLATMC, while HA30 and HA50 revealed significantly more surface roughness, with the HA particles well distributed on the printed surface. Using EDX to determine the ratio of HA on the surface of the 3D-printed templates disclosed limited amounts of HA on the surface of HA10 (Ca atomic ratio = 1.3%). In contrast, much higher amounts of HA were observed on the surfaces of HA30 and HA50 templates, with Ca atomic ratios equal to 21.9 and 36.1%, respectively (Figure 3a). On the other hand, the contact angle measurements of the 3D-printed templates revealed significantly less wettability with the addition of HA up to 30%; HA10 (79.2 ± 2.3) and HA30 (81.4 ± 3.5), compared to that in PLATMC (72.7 ± 3.1) (Figure 3b). However, HA50 (72.6 ± 2.6) had the same contact angle measurement as PLATMC.

With respect to tensile mechanical properties (Figure 3c), the addition of HA to PLATMC, at the presented ratios, significantly reduced the ultimate tensile stress (N/m^2^) of the 3D-printed structures, compared to that in PLATMC (13.2 ± 0.8), where HA50 showed the least tensile stress (7.9 ± 0.3) (Figure 3d). Accordingly, the Young’s modulus of all 3D-printed HA blends was lower than that in PLATMC (409.8 ± 41.3) (Figure 3e).

### In Vitro Degradation and Ca Release Variations among HA Blends

**Figure 4:**
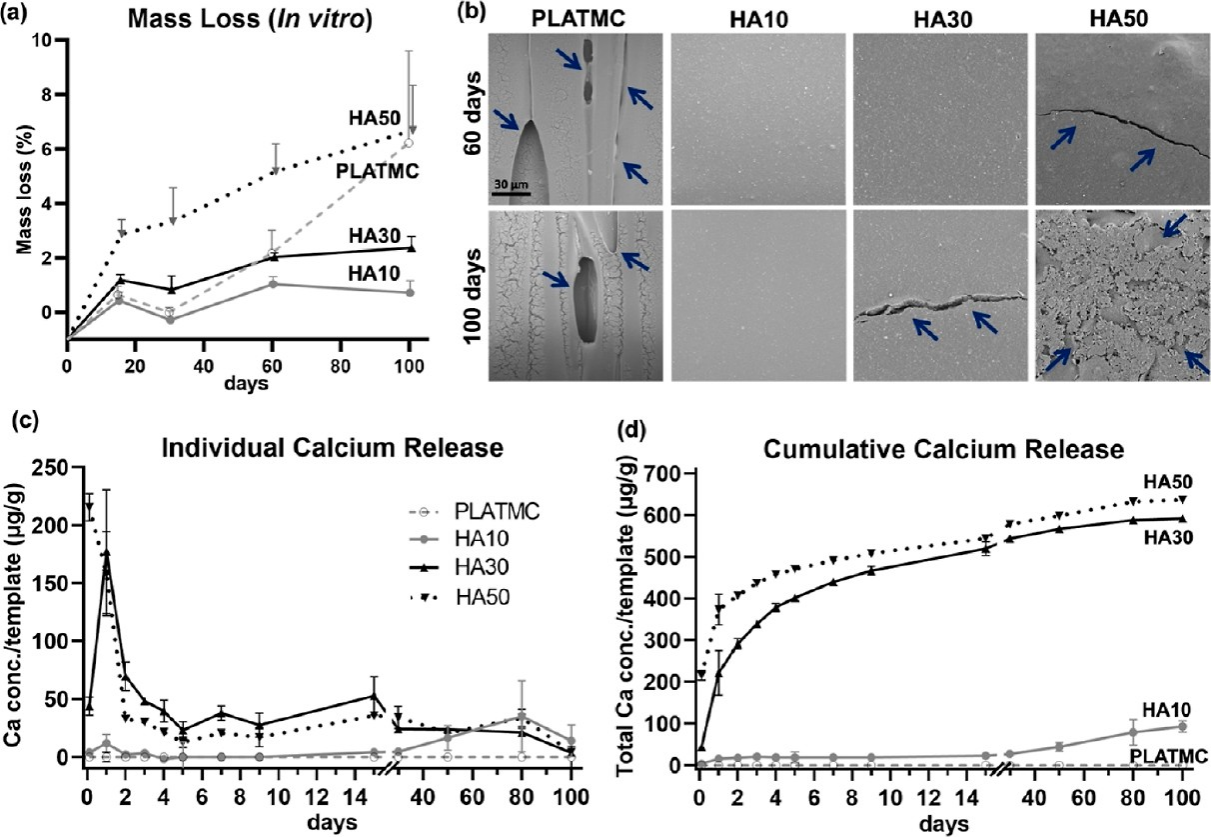
In vitro degradation and Ca release of the 3D-printed HA templates. (a) Line-graph for the mass loss quantification in PBS at 37 °C monitored up to 100 days; (b) SEM micrographs of the printed templates at 60 and 100 days, with signs of degradation marked with blue arrows; and (c,d) line-graphs for the detected in vitro Ca release (in μg/g template) as individual and cumulative amounts, respectively.

In the in vitro degradation assessment, PLATMC did not show mass loss at early time points, 15 and 30 days, but significant mass loss later, from 60 days (2.1% ± 0.8) up to 100 days (6.2% ± 3.3). On the other hand, HA10 underwent minor mass loss even at 60 and 100 days, while HA30 showed significantly higher mass loss than PLATMC only at early time points: (1.1% ± 0.1) and (0.8% ± 0.5) at 15 and 30 days, respectively. However, HA30 exhibited slow subsequent mass loss up to 100 days (2.3% ± 0.4). HA50 exhibited significantly higher mass loss than all the other groups at earlier time points, starting from 15 days (2.8% ± 0.5), with mass loss increasing steadily up to 100 days (6.68% ± 1.65). Thus, mass loss was pronounced only in the PLATMC and HA50 groups, with no intergroup differences at 100 days (Figure 4a). The surface changes monitored by SEM after in vitro degradation at 60 and 100 days showed significant surface erosions and cracks on PLATMC and HA50, as inferred by the mass loss calculations (Figure 4b).

The in vitro Ca release detected from HA blends was calculated and presented in a μg/g template, while the values in μg/mL PBS are presented in Table S1. HA30 and HA50 showed the highest Ca release rates, with initial bursts up to 2 days, of around 290 and 406 μg/g template, respectively, equivalent to 18.9 and 30.4 μg/mL PBS. This was followed by a steady Ca release phase from both groups up to 100 days (Figure 4c). Thus, in general, there was high cumulative Ca release from both HA30 and HA50 up to 100 days, with minor differences between their profiles: total Ca concentration around 591 and 636 μg/g template, respectively, corresponding to 38.6 and 47.5 μg/mL PBS, respectively.

In contrast, HA10 exhibited mild Ca release, at much lower rates, up to 100 days. Limited cumulative Ca release was detected from HA10 up to 30 days: 27.4 μg/g template, equivalent to 1.4 μg/mL PBS, followed by relatively higher Ca release up to 100 days, with a total equal to 92.8 μg/g template, equivalent to 4.8 μg/mL PBS (Figure 4d).

### Templates with High HA Content Exhibited Less Secretion of Mineralized ECM In Vitro

**Figure 5:**
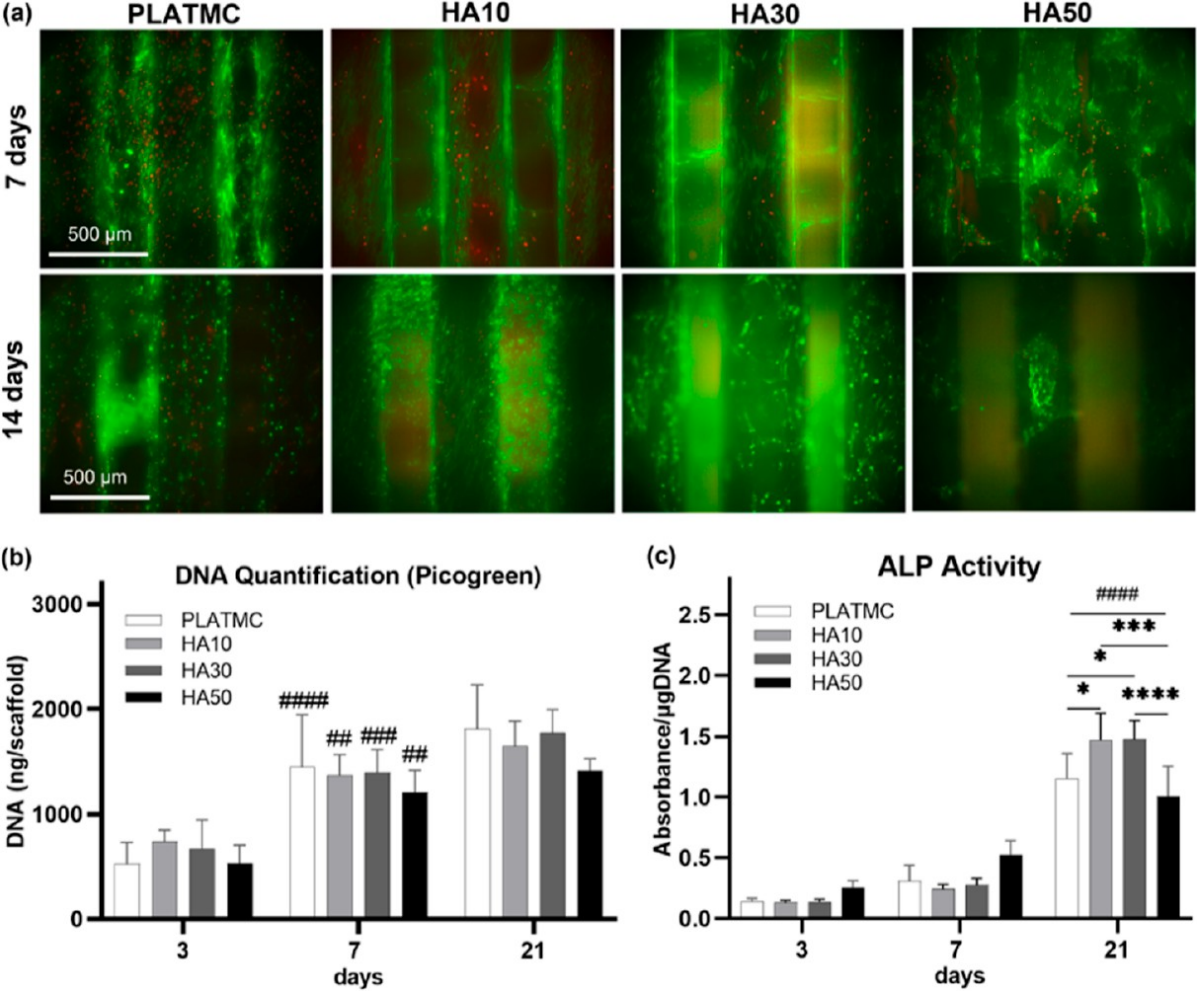
Viability, proliferation, and ALP activity of the seeded hBMSCs on 3D-printed HA blends up to 21 days. (a) Micrographs for live/dead fluorescence staining at 7 and 14 days; (b) column chart of DNA quantification; and (c) ALP activity. Significance between the groups is marked with asterisks (*) at *p* < 0.05; *p > 0.0332, **p > 0.0021, ***p > 0.0002, and ****p < 0.0001. Statistical significance between each time point and the previous time point in the same group is marked with hash symbol (#).

No intergroup differences were observed among the cultured hBMSCs in vitro on PLATMC and HA blends, in terms of the seeding efficiency (Figure S1a) and cellular activity detected by alamarBlue (Figure S1b) at 3 and 7 days. This was in accordance with the pictured live/dead stained samples at 7 days, where no obvious intergroup differences could be noted. Nevertheless, at 14 days, fewer viable cells were observed attached to HA30 and much less to HA50, compared to PLATMC and HA10 groups (Figure 5a). In addition, a lower proliferation rate, measured by quantified DNA, was noted for HA50 at 21 days (Figure 5b).

**Figure 6:**
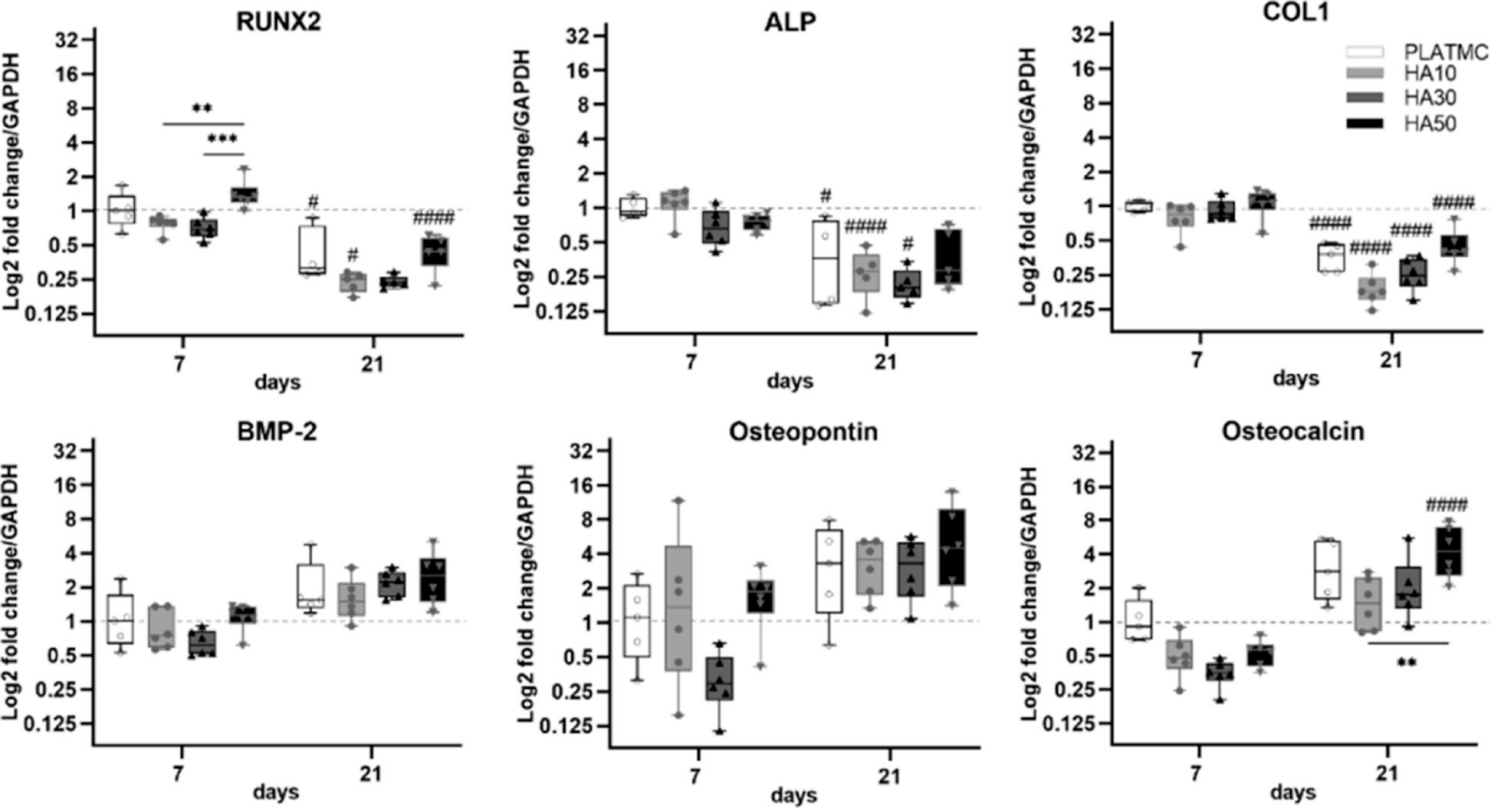
Box plots of the osteogenic gene expression of seeded cells, diverse markers at 7 and 21 days. Significance between the groups is marked with asterisks (*), at *p* < 0.05; according to Bonferroni correction adjustment (instead of Tukey post hoc), due to higher data variances. *p > 0.0332, **p > 0.0021, ***p > 0.0002, and ****p < 0.0001. Statistical significance between each time point and the previous time point in the same group is marked with hash symbol (#).

Some early signs of osteogenic differentiation were noted for HA50, higher than that for HA10 and HA30, including ALP activity as early as 3 and 7 days (Figure 5c) and the expression of RUNX2 at 7 days (Figure 6). However, this was reversed at 21 days, where HA50 had the lowest ALP activity and HA10 and HA30 exhibited markedly higher ALP activity. Otherwise, there were no relevant intergroup differences in the early osteogenic gene markers (RUNX2, ALP, and COL1) or the intermediate to late markers (BMP-2, Osteopontin, and Osteocalcin), expressed by the seeded cells on PLATMC or HA blends (Figure 6).

**Figure 7:**
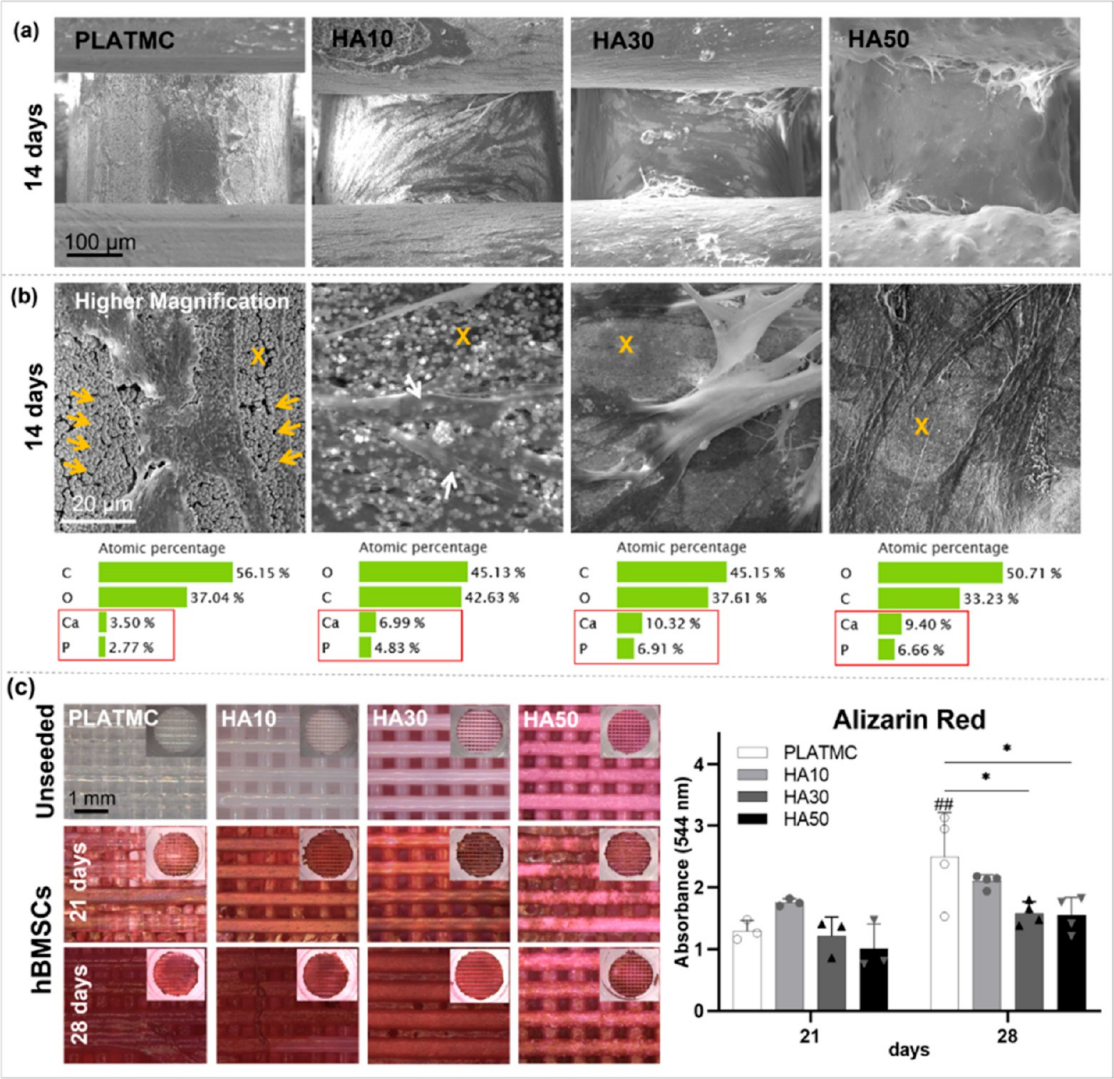
Summary of mineralized ECM characterization. (a) SEM at 14 days showing cellular attachment and ECM secretion; (b) SEM at higher magnification showing the secreted ECM, with globular accretions (marked with yellow arrows) on PLATMC, while calcified collagen (marked with white arrows) is shown on HA10. In addition, EDX shows the atomic percentage of Ca and P contents presented on the surface (marked with yellow ×). (c) Micrographs of Alizarin red-stained 3D-printed PLATMC and HA templates seeded with hBMSCs at 21 and 28 days (scale bar = 1 mm), compared to unseeded templates (blank), with inset pictures for the overall stained templates and a column chart showing their quantification (optical density) at 544 nm (absorbance). Significance between the groups is marked with asterisks (*) at *p* < 0.05; *p > 0.0332.

However, less mineralized ECM secretion was noted in vitro in the HA30 and HA50 groups, compared to that in PLATMC, confirming the variations in live/dead stain at 14 days. When ECM secretion was assessed by SEM at 14 days (Figure 7a), the PLATMC samples were covered with attached cells secreting mineralized globular accretions of the cement line matrix, while a denser mineralized collagen matrix was noted on HA10. In contrast, higher magnifications revealed much less mineralized ECM on HA30 and less again on HA50 (Figure 7b). Moreover, surface analysis of the ECM by EDX (Figure 7b) disclosed higher Ca and P ratios on PLATMC and HA10, compared to their controls before culturing the cells (compared to Figure 3a). In contrast, much lower Ca and P ratios were disclosed on the surfaces of HA30 and HA50, compared to their controls before culturing the cells (compared to Figure 3a).

These observations were in accordance with the biomineralization assay, stained with Alizarin red and quantified: HA10 showed higher calcified matrix than PLATMC and other HA blends at 21 days (Figure 7c). However, at 28 days, an obvious boost in biomineralization was seen in pristine PLATMC and HA10, while it was statistically the lowest in HA30 and HA50.

### Templates with Low Percentages of HA Exhibited Greater Bone Regeneration

**Figure 8:**
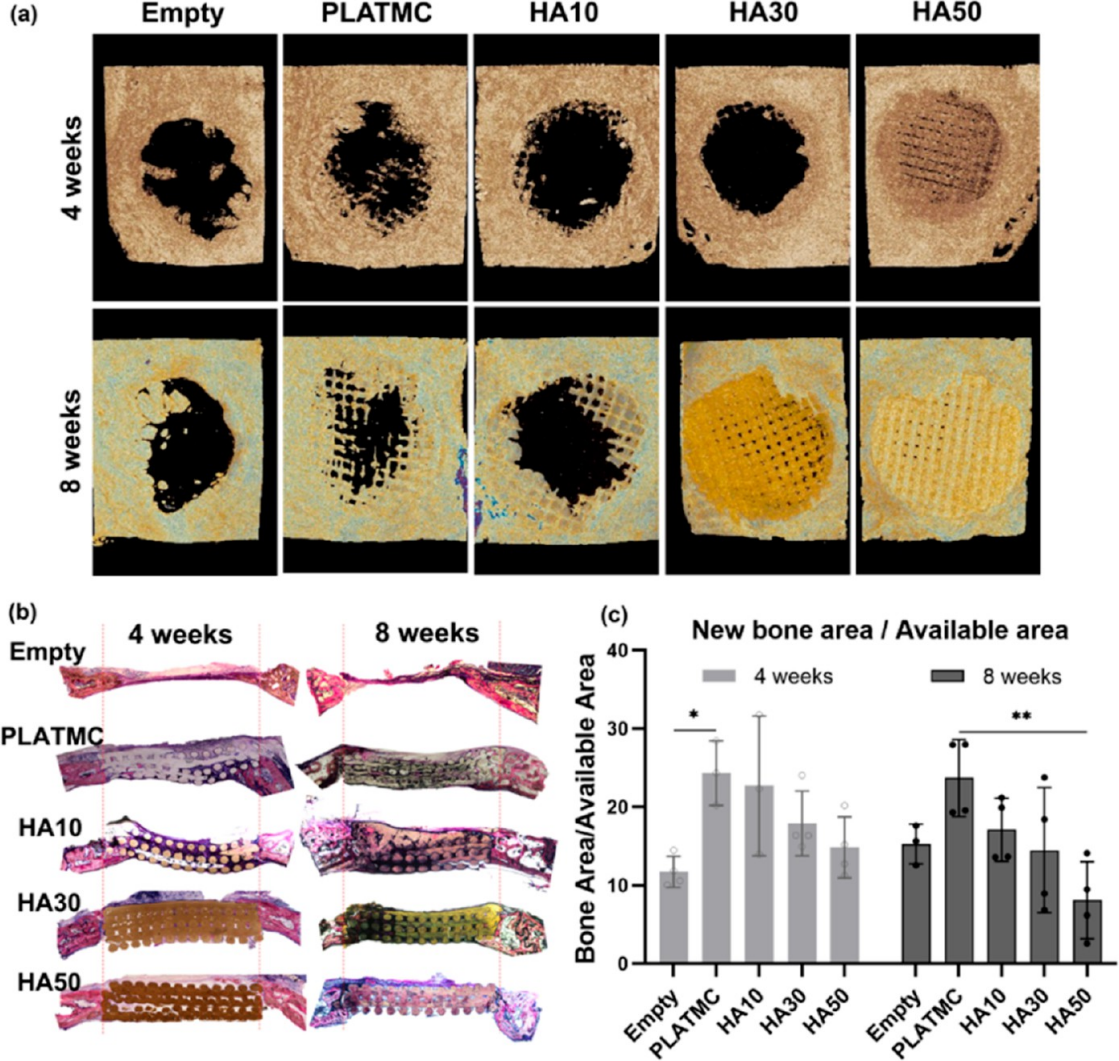
Summary of the bone tissue engineering impact of the implanted 3D-printed HA templates in the calvarial bone defect (CBD) model in rabbits. (a) Reconstructed μCT pictures of the implanted templates in CBDs; (b) representative nondecalcified histological sections of CBDs at 4 and 8 weeks, stained with Toluidine blue and acid fuchsin; and (c) bar chart of the histomorphometric analysis of new bone area per total available area (NBA/ADA). Significance between the groups is marked with asterisks (*) at *p* < 0.05; *p > 0.0332 and **p > 0.0021.

In the CBD model, the reconstructed μCT pictures of the implanted templates showed some intergroup differences after 4 and 8 weeks (Figure 8a). However, it was difficult to interpret the HA30 and HA50 templates because their radiographic densities were so similar to that of the surrounding bone. Thus, no quantitative data are shown for μCT results. In general, some bone ingrowth toward the defect center was obvious on PLATMC and HA10, following the scaffold strands from all around the defect margins. Meanwhile, small amounts of bone were also observed creeping in from the margins of the empty defects. For accurate histomorphometric analysis, the area of new bone, in relation to the total available defect area, was quantified from nondecalcified histological sections (Figure 8b). The calculated NBA at 4 weeks disclosed that compared to HA30 and HA50, PLATMC and HA10 had the greatest amount of new bone, and for PLATMC, this was statistically significantly higher than the empty defects (Figure 8c). Moreover, the same trend was observed at 8 weeks: less NBA was quantified in HA blends with higher HA ratios. Thus, HA50 showed the least NBA at 8 weeks.

**Figure 9:**
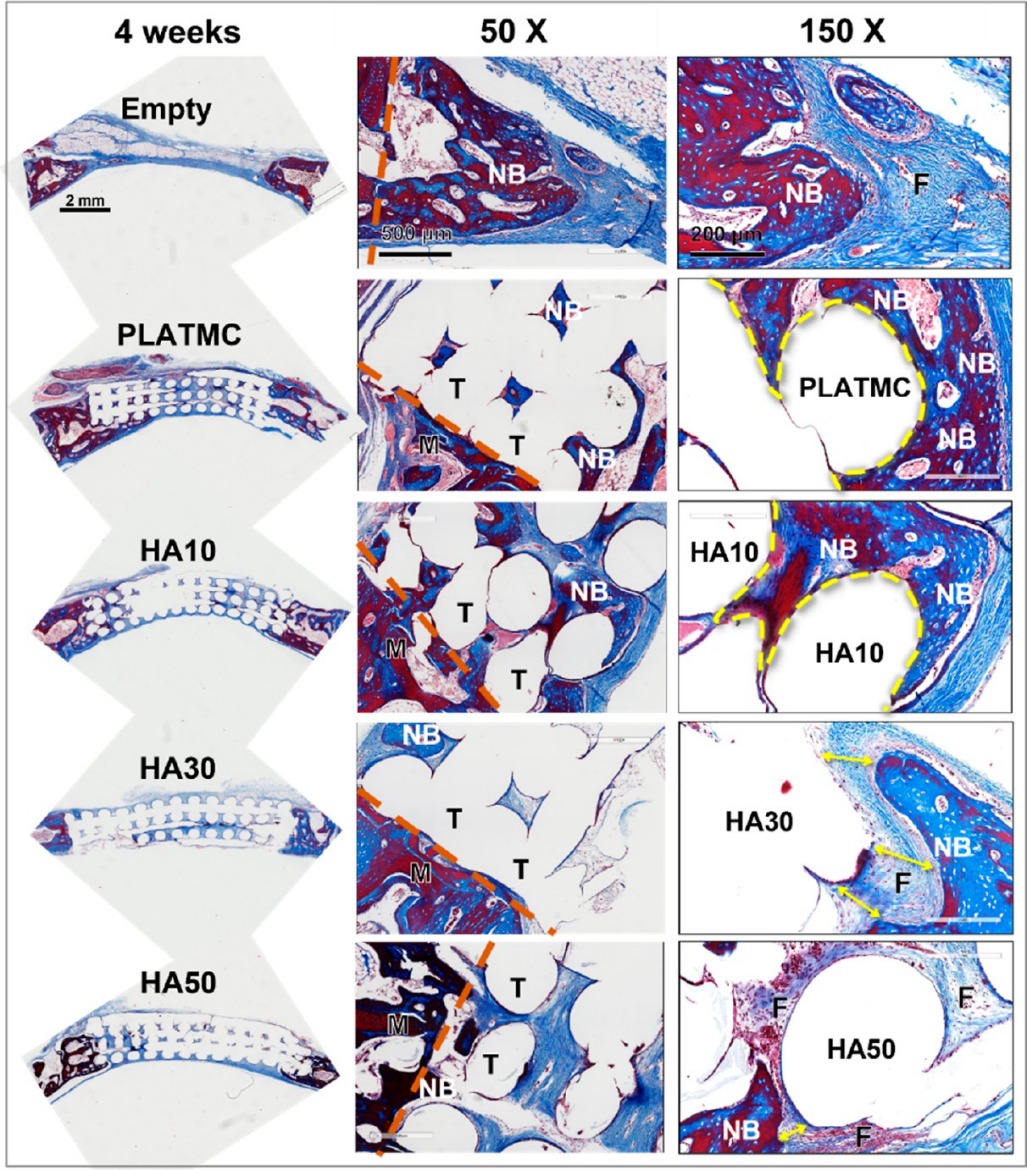
Representative decalcified histological micrographs of the CBDs containing the implanted 3D-printed templates at 4 weeks and two higher magnification views of bone ingrowth, 50× and 150×, stained with Masson’s trichrome. At 50x: brown dashed line marks the interface between (M) and (T); (M) represents the original margin of the defect; (T) represents the implanted template; and (NB) represents the new bone area. At 150×: curved, yellow dashed line marks the characterized NB contact line with T at higher magnifications (on PLATMC and HA10); (yellow double arrow marks the characteristic gap between NB and T (at HA30 and HA50); and (F) indicates the fibrous connective tissue interface.

Histological examination of decalcified sections at high magnifications showed that in the empty defects (negative controls), marginal bone was undergoing remodeling toward healing the created defect (Figure 9). The remodeled bone creeping into the empty defects was very small in quantity, however, and always accompanied by thinning of the original bone margins surrounding the defect.

**Figure 10:**
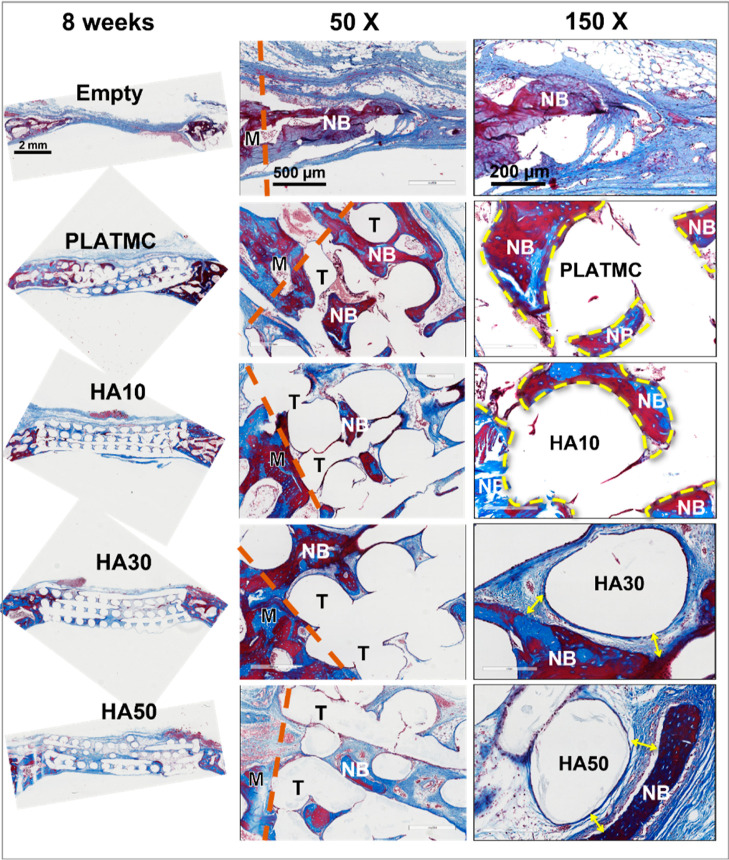
Representative decalcified histological micrographs of the CBDs containing the implanted 3D-printed templates at 4 weeks and two higher magnifications, 50× and 150×, of the bone ingrowth, stained with Masson’s trichrome. At 50×: brown dashed line marks the interface between (M) and (T); (M) represents the original margin of the defect; (T) represents the implanted template; (NB) represents the new bone area. At 150×: curved, yellow dashed line marks the characterized NB contact line with T at higher magnifications (PLATMC and HA10); yellow double arrow marks the characteristic gap between NB and T (HA30 and HA50); and (F) indicates the fibrous connective tissue interface.

In contrast, in the defects implanted with 3D-printed PLATMC and HA blends, HA10 exhibited osteoconduction and contact osteogenesis comparable with that of PLATMC, with spots of active bone formation integrated onto the surface of the HA10 strands. No contact osteogenesis was noted on HA30 and HA50 surfaces, and in most cases, only fibrous connective tissue could be observed attached to their surfaces (Figure 9). At 8 weeks, no additional histological changes were recorded with respect to either the quantity of bone formation or its contact with the template surface (Figure 10).

## Discussion

The proposed mechanism of osteoconductivity and new bone formation on HA surfaces was related to their Ca release, which facilitates biomineralization.^15,26^ However, the exact role of the released local Ca concentrations in relation to osteoconduction has not been studied extensively. In addition, others reported amplified inflammatory response in vivo associated with increased local extracellular Ca concentrations.^27^

In this study, PLATMC was selected as the base polymer because of its promising osteoconductive applications.^28−30^ The methods of preparing HA blends and the 3D-printing used in this study were intended to fabricate homogeneous, reproducible porous HA templates, with accurate HA bulk ratios. Although the study disclosed reduced tensile properties of HA templates compared to those of pristine PLATMC, the melt-extrusion 3D-printing procedure offered an advantage over the photo-cross-linked polymer-based templates, represented as a high degradation rate, reported to be about a 100-fold higher than that observed in photo-cross-linked templates.^31^ Processing techniques without cross-linking have been recommended to facilitate the fabrication of degradable and osteoconductive templates for bone tissue engineering.^24^

On the other hand, the 3D-printed HA10, HA30, and HA50 templates exhibited different surface concentrations of HA, and their Ca release varied accordingly. Consequently, the differentiating seeded cells were exposed to three different conditions/levels. HA10 exhibited mild initial release of Ca, followed by very limited amounts up to 30 days: 15 μg/g on day 1 and 27 μg/g after 30 days. In contrast, HA30 and HA50 exhibited high initial Ca release (221 and 373 μg/mL, respectively, on day 1) followed by continuous release of considerable amounts up to 30 days.

With respect to biological effects, HA10, with mild to limited Ca release, had no relevant effect on cell proliferation but achieved the hypothesized enhancement of osteoconduction. This was evidenced as abundant calcified ECM as early as 14 days and high ALP activity at 21 days, along with greater amounts of mineralized matrix, detected by Alizarin red stain, compared to PLATMC. On the other hand, HA30, which exhibited high initial and considerable continuous Ca release, expressed the same level of osteogenic markers and ALP activity as HA10 but much less calcified ECM production at 14, 21, and 28 days.

In contrast, HA50 exhibited an initial release burst of Ca, followed by continuous release of considerable amounts of Ca and reduced cell proliferation, demonstrated by live/dead stain and quantified DNA, and also revealed early higher osteogenic differentiation markers (RUNX2 expression and ALP activity at 7 days). However, no adequate mineralization was observed on its surface. Instead, ALP activity of HA50 was significantly reduced, and at 21 days, HA50 exhibited clearly less production of mineralized ECM than HA10 and HA30. This could be explained by the reported gradual increase in cytotoxicity correlated with Ca concentrations of 50 up to 500 μg/mL.^17^

Our results are in accordance with those reported for photo-cross-linked HA templates in the reviewed literature, where the two fabricated HA blends (HA 20 and 40%) exhibited mild continuous Ca release in vitro, which did not exceed 35 μg/template up to 30 days.^24^ In the latter study, both HA blends exhibited improved in vitro osteoconduction (Alizarin red staining at 28 days) and higher amounts of bone tissue ingrowth when implanted in rat calvarial defects, compared to plain polymer templates. No significant intergroup differences in osteoconductivity were observed between HA 20% and HA 40% in vitro or in vivo.^24^

At 14 days, EDX comparison of the Ca present on each HA template surface before and after seeding with hBMSCs revealed a significant reduction in Ca concentrations on HA30 and HA50 surfaces, with no obvious calcified matrix deposition. In contrast, much higher Ca concentrations were present on PLATMC and HA10 surfaces, related to the deposition of calcified matrix due to the consistent osteoconduction process.^32^ However, the limited degradation exhibited by HA10 templates in the current study indicated that HA inclusion, at this reduced ratio, acts as a space filler, which reduces internal water absorption. This coincided with the increased contact angle values compared to the plain PLATMC templates. However, this was not ideal for the ultimate aim of bone tissue engineering: degradation of the implanted templates should not start until after bone formation, in order to provide a secondary space for bone modeling.^33^

In the induced calvarial defects in rabbits, bone ingrowth into the defects was observed in all groups, including the empty defect group. This was in accordance with previous studies in rats and rabbits, where the empty defects showed hypo-mineralized, remodeled bone margins creeping into the created critical size defects.^14,34^ On the other side, the original defect bone margins of the empty defects showed thinning or reduction in volume, which was not observed in all template implanted groups, that supported and integrated with the original defect margins.

The results of 3D-printed HA blends implanted in the calvarial defect model were in accordance with the in vitro outcomes: in vivo, osteoconduction occurred only under conditions that caused a mild Ca release in vitro. The implanted HA10 showed new bone ingrowth and contact osteogenesis as high as for PLATMC. On the other hand, HA30 and HA50, which in vitro exhibited high/burst initial Ca release followed by considerable continuous release, exhibited significantly less bone ingrowth in vivo, and a larger fibrous tissue layer was observed separating the template surface from the adjacent bone ingrowth. This could be defined as distance osteogenesis, away from the template surface, a characteristic feature of poor osteoconductive templates.^35,36^

## Conclusions

The results of the study confirm that the rate of Ca release is a critical factor for enhancing osteoconduction by CaP-templates and should be considered into the design and evaluation of osteoconductive templates. To promote osteoconduction, the inclusion of HA in non-cross-linked polymeric templates should be undertaken with caution: Ca release should be controlled and should not exceed 25–30 μg/g template. Both in vitro and in vivo, Ca released at initial or continuously high concentrations inhibits osteoconduction.

## Supporting Information Available

Additional cumulative Ca release concentrations, seeding efficiency, and cellular viability results (PDF)
