# Supplementary material for: Correlation between Ca Release and Osteoconduction by 3D-Printed Hydroxyapatite-Based Templates
Source: ACS Appl Mater Interfaces. 2024 May 25;16(22):28056–69. doi: 10.1021/acsami.4c01472 (PMC11163400; doi:10.1021/acsami.4c01472)
Supplement: Supplementary file 1 — am4c01472_si_001.pdf [file am4c01472_si_001.pdf]

# Supporting Information

## Correlation between Ca release and osteoconduction by 3D-printed hydroxyapatite-based templates

Mohamad N. Hassan[*1,2], Ahmed M. Eltawila[3,4], Samih Mohamed-Ahmed[1], Wessam M. Amin[3], Salwa Suliman[1], Sherif Kandil[3], Mohammed A. Yassin[1,5], and Kamal Mustafa[*1]

[1] Centre for Translational Oral Research (TOR), Department of Clinical Dentistry, Faculty of Medicine, University of Bergen, Årstadveien 19, 5009 Bergen - Norway.

[2] Orthopedic Clinic, Haukeland University Hospital, Helse Bergen, Haukelandsveien 28, 5021 Bergen - Norway.

[3] Department of Materials Science, Institute of Graduate Studies and Research (IGSR), Alexandria University, El-Shatby, 21526 Alexandria - Egypt.

[4] Department of Dental Biomaterials, Faculty of Oral and Dental Medicine, Delta University for Science and Technology, Coastal International Road, 11152 Gamasa - Egypt.

[5] Biomaterials section, Department of Clinical Dentistry, Faculty of Medicine, University of Bergen, Årstadveien 19, 5009 Bergen - Norway.

**Corresponding Authors' contact:**

- Prof. Kamal Mustafa, University of Bergen. kamal.mustafa@uib.no

Tel: (+47) 55586097

- Dr. Mohamad Nageeb Hassan, University of Bergen. nageeb.hassan@uib.no

Tel: (+47) 55586382

**Supporting Information**

**Table S1**: Cumulative Ca release concentrations represented in µg/g template and the equivalent concentrations calculated in µg/ml PBS

| | Cumulative Ca concentrations µg/ml PBS | | | | Cumulative Ca concentrations µg/g template | | | |
|---|---|---|---|---|---|---|---|---|
| **Days** | **PLATMC** | **HA10** | **HA30** | **HA50** | **PLATMC** | **HA10** | **HA30** | **HA50** |
| **0** | -2.4E-06 | 0.202198 | 2.864268 | 16.12302 | -4.8E-05 | 3.848967 | 43.86321 | 215.5484 |
| **1** | -7.3E-06 | 0.820414 | 14.44127 | 27.96219 | -0.00015 | 15.61712 | 221.1527 | 373.826 |
| **2** | -9.2E-06 | 0.933365 | 18.99286 | 30.41508 | -0.00018 | 17.76722 | 290.8555 | 406.6187 |
| **3** | -8.3E-06 | 1.102099 | 22.14161 | 32.65184 | -0.00017 | 20.97917 | 339.0752 | 436.5219 |
| **4** | -1.2E-05 | 1.004481 | 24.72047 | 34.25084 | -0.00024 | 19.12096 | 378.5677 | 457.8989 |
| **5** | -1.2E-05 | 1.004478 | 26.21721 | 35.20652 | -0.00024 | 19.1209 | 401.4887 | 470.6754 |
| **7** | -1.2E-05 | 1.004474 | 28.6808 | 36.74975 | -0.00024 | 19.12082 | 439.2159 | 491.3068 |
| **9** | -1.2E-05 | 1.004478 | 30.46621 | 38.00711 | -0.00024 | 19.1209 | 466.5575 | 508.1165 |
| **15** | -1.3E-05 | 1.21272 | 33.90779 | 40.67615 | -0.00027 | 23.08491 | 519.2618 | 543.7988 |
| **30** | -1.7E-05 | 1.440482 | 35.47426 | 43.22713 | -0.00034 | 27.42052 | 543.2505 | 577.9028 |
| **50** | -1.3E-05 | 2.305066 | 37.02121 | 44.76664 | -0.00026 | 43.87844 | 566.9404 | 598.4845 |
| **80** | -1.5E-05 | 4.146712 | 38.40361 | 47.29112 | -0.00031 | 78.93538 | 588.1104 | 632.2342 |
| **100** | -1.8E-05 | 4.877574 | 38.62884 | 47.59837 | -0.00037 | 92.8478 | 591.5596 | 636.3418 |

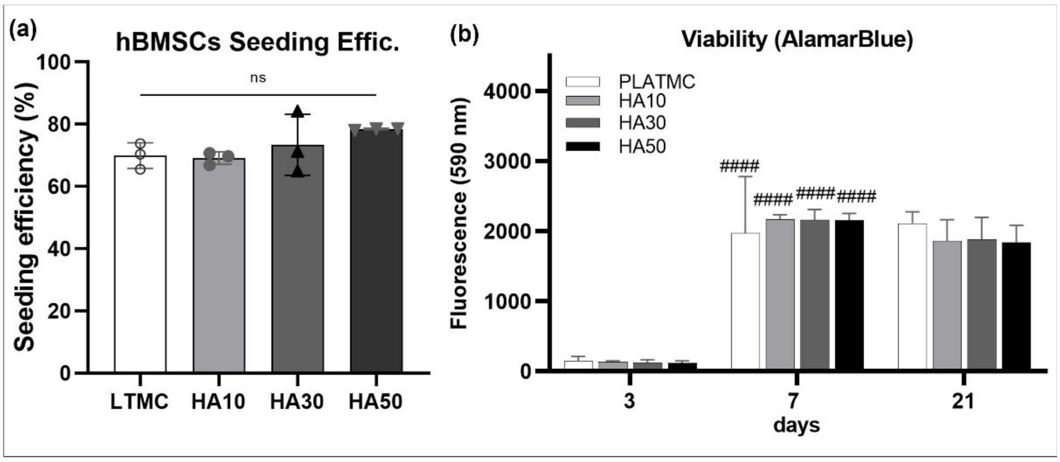


**Figure S1**: (a) Seeding efficiency after 8 hours, (b) alamarBlue assay of cellular activity at 3, 7 and 21 days. No significance between the groups was recorded at each time point at $p < 0.05$. Statistical significance between each time point and the previous time point in the same group is marked with a hash symbol (#).

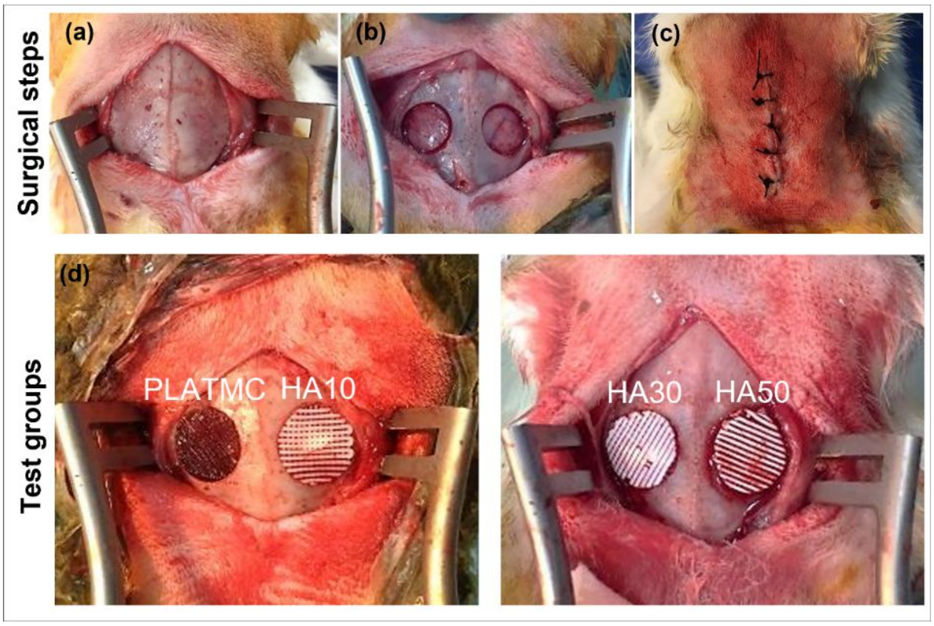


**Figure S2**: (a-c) surgical procedures to create calvarial bone defects, (d) shape of the test groups (PLATMC and HA blends) implanted in the calvarial bone defects.
